# Children left unattended in parked vehicles: a focus on recent italian cases and a review of literature

**DOI:** 10.1186/1824-7288-39-71

**Published:** 2013-11-06

**Authors:** Pietro Ferrara, Flaminia Vena, Olga Caporale, Valentina Del Volgo, Pio Liberatore, Francesca Ianniello, Antonio Chiaretti, Riccardo Riccardi

**Affiliations:** 1 Institute of Pediatrics, Catholic University of Sacred Heart, Rome, Italy; 2 Campus Bio-Medico University, Rome, Italy

**Keywords:** Child, Hyperthermia, Vehicles, Injury

## Abstract

Every year a lot of children die from heat stroke and hyperthermia because they have been left unattended in closed automobiles. These fatalities have augmented slightly in the past decade, but they are a poor recognized type of vehicle injury and little research has been done to discover the circumstances surrounding the fatal event. Two factors make children more prone to hyperthermia than adults: children have a greater surface area body mass ratio than adults and their thermoregulation is less efficient than adults. A scientific explanation about how it can happen that a parent leaves his child unattended in the car could be related to the Working Memory (WM): stress increases interference from irrelevant information, impairing selective attention and WM and influencing behavior. In the last two years in Italy 16 cases of child hyperthermia due to abandonment in motor vehicle were identified. These findings suggest that educational programs and literature for parents regarding automobile safety should include information about the potential dangers of heat stress, in fact, as these events are mostly unintentional, legislative efforts may be vain.

## Introduction

Injuries in stationary vehicles for children younger than 14 years old are poorly recognized type of vehicle injury and receive far less attention than motor vehicle crashes. In fact, according to data of the CDC (Centers for disease Control and Prevention), on average 12,175 children from 0 to 19 years of age die each year in the United States from an unintentional injury and the main cause is transportation (most of them because of motor vehicle traffic-related injuries) [1]. Although this information is important, a lot of children die from heat stress because they have been left in closed automobiles, but few data regarding the circumstances surrounding the fatal event are available. Previous researches confirm that the leading cause of death for children is stroke and hyperthermia after being left unattended in motor vehicles. Recent studies listed the total number of deaths caused by hyperthermia in the United States and identified 556 cases from the 1998 up to 2012, with 29 cases reported in 2012 and 33 cases reported in 2011. On average 38 is the number of child hyperthermia fatalities per year [2]. It is interesting pointing out that there are remarkable differences among geographic distribution, circumstances, victim characteristics (male or female subjects) and among children and adults: children seems to be more prone to develop hyperthermia when inside a closed, hot vehicle [3]. Two factors make children more prone to hyperthermia than adults: children have a greater surface area body mass ratio than adults and a child’s thermoregulation is less efficient than adults [4]. A study conducted to investigate the thermoregulation of young children compared to that of adults points out that the sweating and skin temperature responses of the children are not enough to prevent a rise in body temperature [5]. These would suggest that the thermoregulation system is immature and that young children have the disadvantage of heating up easily due to their smaller body sizes [5]; in addition the potential physical and physiological differences, adults have the ability to modify their behavior on the basis of the environment. They can undergo cooling measures: take off excess clothing, obtain cold drinks and seek a cooler environment (getting out of a hot car) [4].

The aim of this study is to summarize the circumstances surrounding the fatal event, highlighting the major causes of children death and injuries, trying to explain how it could happen that a caregiver forgets his child and underscoring the need for effective interventions. Furthermore 16 cases of illness or death of children caused by the fact of being left unattended in parked vehicles were identified in Italy.

## Methods

Data collected by Null of the San Francisco State University (Department of Geosciences), updated the 25^th^ September, 2012, have been used to identify hyperthermia (heat stroke) deaths of children since 1998 in the United States [2]. This publicly available database is updated daily and it is the most complete child hyperthermia mortality database [3]. It contains information on the number of deaths, age group, circumstances, and describes the vehicle heating dynamics.

A second source was a separate internet search for children left by their mother or father in parked vehicles occurring in Italy between 1^st^ May, 2011 and the 31^st^ August, 2012, using newspaper indexes, news websites and internet searching engines such as Google. Keywords related to heat, motor vehicles, hyperthermia, child, abandonment and enclosure were used. 16 cases were collected and children were defined as aged ≤11 years but in 14 out of 16 cases children were aged ≤3 years. All cases reported occurred in Italy and in our study were enclosed all children (males and females), regardless of the outcome. We reported whether children left unattended were intentionally left unattended or not and if the case was unclear. We also looked at where it happened, the cause of the “abandonment” and for how long, the child’s age and the outcome.

## Results

Between the 1^st^ of May 2011 and the 31^st^ of August 2012, we identified 16 cases of child hyperthermia due to abandonment in motor vehicle. Table 1 illustrates the circumstances surrounding the events. As shown in the table, we identified 7 males and 5 females and in 4 cases we could not identify the gender of the child. In 3 cases the children left unattended were 2. In 12 out of 16 (75%) cases the children were left intentionally in the car, in 3 out 16 (18%) unintentionally.

**Table 1:** **Cases of children left in vehicles in Italy between May 1**^**st **^**2011 and August 31**^**st **^**2012**

**Date**	**Gender**	**Age**	**Time**	**Caregiver**	**Circumstances**	**Intentionally**	**Outcome**
18/05/2011	Female	22 months	6 h	Father	Go to work	No	Death
23/05/2011	Unknown	1 year; 3 years	30 min	Both parents	Do the shopping	Yes	Positive
24/05/2011	Male	3 months	20 min	Both parents	Go shopping	Yes	Positive
27/05/2011	Male	11 months	3 h	Father	Go to work	No	Death
02/06/2011	Male	3 years	2 h	Mother	Go to a party	Yes	Positive
03/06/2011	Male	3 years	2 h	Mother	Go to a party	Yes	Positive
04/06/2011	Unknown	2 11-month-old twins	Undetermined	Both parents	Play with the slot machines	Yes	Positive
13/06/2011	Female	2 years	Undetermined	Mother	Go clubbing	Yes	Positive
06/08/2011	Male	13 months	40 min	Both parents	Take a walk	Undetermined	Positive
28/02/2012	Female	3 years	3 h	Driver of the school bus	Forget the child in the bus	No	Positive
24/05/2012	Male	8 months	Undetermined	Both parents	Do the shopping	Yes	Positive
21/07/2012	Male	2 years	1 h 30 min	Mother	Go shopping	Yes	Unknown
13/08/2012	Females	4 years; 11 years	2 h	Both parents	Go shopping	Yes	Positive

## Discussion

Our results are in contrast with Booth et al’s study of 2010 that collected cases between 1999 and 2007 and identified 192 cases of children left unattended: 25 (13%) were left unattended intentionally, 145 (75.5%) were unintentionally left unattended and in 22 (11.5%) cases the intentions were unclear [3]. Data collected by Null had similar findings: he reported that 494 children died from vehicular hyperthermia for a thirteen year period (1998 through 2011) and he showed that 52% were “forgotten” by caregiver and 17% intentionally left in vehicle by adult [2].

Concerning our study in 13 out of 16 cases the outcome was positive. The mother was responsible for the child at the time of the event in 4 cases, the father in 2 cases and both parents in 9 cases. When evaluating the caretakers liability, there were 18.7% cases where the children were simply forgotten in the car or the caretaker forgot to drop the child off at daycare. Additionally 75% of parents left the child intentionally to go clubbing, to reach their job, to go to the market or to play slot machines in a bar. In a survey performed by Roberts and Roberts, approximately one quarter of interviewed women admitted they left their child in unattended cars [6]. In a survey performed by Guard and Gallagher, circumstances surrounding the fatal event were defined: 27% of deaths (46/171) were represented by children who gained access to unlock vehicles and died while playing. 73% of deaths (125/171) occurred when adults left children unattended in vehicles [7]. Furthermore unattended children tended to be younger than children who died while playing. Three quarter of our cases occurred during warmer months: spring and summer.

According to these data, one might well ask how it can happen that a parent leaves his child unattended in the car. A scientific explanation could be related to the Working Memory (WM). WM refers to the system or systems that are assumed to be necessary in order to keep things in mind, while performing complex tasks such as reasoning, comprehension and learning [8]. There is great evidence that stress and enhanced glucocorticoids levels can influence memory performance with both negative and positive consequences. A recent study assessed the effects of stress and cortisol on a variety of memory tasks in male human subjects and demonstrated that there is a stress-induced working memory impairment [9]. These could explain how stress and busy everyday life could influence our behavior and bring a parent to leave unattended his child in the car.

Sun rays penetrate the glass window of a vehicle, striking the interior and heating exposed surfaces. If the windows are closed, the heat cannot escape and the car will continue to get hot throughout the day. Even on days when the weather is cool but sunny, the interior of the vehicles heats rapidly [10]. Furthermore it was reported that objects such as dashboard, steering wheel and child seat heat the adjacent air (reaching temperatures in the range of 180 to over 200°F) by conduction and convection [4]. Previous studies examined the internal environment of motor vehicles [6].

King et al. indicated that there was a rapid rise in interior temperature with at least 75% of the maximum stabilized temperature being reached within five minutes of closing the doors and maximized within 15 minutes to 51 to 67°C (124-153°F) with significant differences depending on the particular vehicles characteristics [11]. Opening the windows 20 cm had minimal effect on the temperature rise and maximum temperature attained. These results are similar to previous data, that ascribed to the vehicle interior color the biggest determining factor and to the first 10 minutes the most important increase in temperature.

McLaren et al. had similar results. In their study 80% of the temperature rise occurred in the first 30 minutes obtaining a maximum at 60 minute [4].

Bouchama and Knochel demonstrated that the first manifestation of heat illness is heat stress, a physical and physiologic strain, next is exhaustion characterized by dehydration and a core temperature reaching 37°C to 40°C and finally heat stroke. Research performed in the past decade has shown that heat stroke results from thermoregulatory failure and an increased acute-phase response with altered expression of heat-shock proteins. Heat stroke is defined clinically as a core body temperature that rises above 40°C and that is accompanied from hot, dry skin and central nervous system abnormalities such as convulsion, delirium or coma [12]. Previous studies confirmed that children are more prone to heat illness than adults for several reasons. Tsuzuki-Hayakawa and Tochihara demonstrated that children aged between 9 months to 4.5 years and placed for 30 minutes in a room of 35°C, although having a smaller body mass, had a rectal temperature that increased more rapidly and was higher than mother’s [5]. Fluid loss from sweating is the major initial physiologic imbalance and exposure to light and radiant heat could double this base level [13]. Increase in ambient temperature and activities such as crying or restlessness would also cause an increased fluid loss [14,15]. Kuno reported that sweat secretion is conditioned by the excitability of the sweat centers and that sweating for the children was shorter than for the mothers, although the activity of a single sweat gland was only about half or one-third that of the mother [16].

It would be interesting to discover how long until the infant experiences adverse effects from a particular heat, but it is difficult to study for ethical reasons [4].

The findings in this report are consistent with other researches that indicate that children left unattended in motor vehicles are at an increased risk of injury and death.

Leaving a child alone in a car can be considered a form of neglect. As according to The Child Abuse and Prevention Treatment Act (CAPTA), child abuse and neglect are defined as: “any recent act or failure to act on the part of a parent or caretaker, which results in death, serious physical or emotional harm, sexual abuse, or exploitation, or an act or failure to act which presents an imminent risk of serious harm”. Additionally, what is considered neglect varies based on the age and the developmental level of the child: for instance leaving a child unattended for an hour is considered neglect when the child is young, but not when the child is a teenager [17].

Several areas for possible intervention include education, legislation and regulation. Parents and caretakers should be warned and exhorted not to leave children alone in motor vehicle even for just a few minutes. How often is the quick 10 minutes visit to the supermarket, with children left in the parked car, then converted to 30/40 minutes?

Special precaution should be taken with small infants and children during milder and even sunny days, in order to prevent the “greenhouse effect” occurring rapidly in a car where the windows are transparent to solar radiation but opaque to long-wave radiations. Jasni and Nasir found out the tinted windows as an overall good performance in reducing the maximum temperature inside the car because they decrease the transmitted radiations trough the windows [18]. As recommended, people have to make “look before you leave” a routine whenever getting out of the car or to place their purse or briefcase in the back seat as a reminder that they have a child in the car.

Parents could also do something like purchasing a booster seat, writing a note or keeping an object such as a stuffed toy where the driver will notice it before leaving the car or asking the childcare provider to call if the child is not stand out for childcare and avoid to park in a sunny place. However, many parents may not conceive of forgetting their child in a car and even with an incentive could not purchase a system. For this reason a technological solution in which a sensor alerts a caregiver that a child has been left in a car will be the only plausible intervention strategy.

## Conclusions

According to the 2001–2010 Italian Status Report on Road Safety, road deaths result reduced by 43% in 10 years, after the creation of the “Italian Road Safety Awareness Campaign” started in 2001 with the aim of promoting safety belt use and compliance with speed limits to reduce injuries and deaths from traffic crashes. Consequently we propose the scientific community to start a “Children deaths in parked vehicles awareness Campaign”.

Educational programs and literature for parents (parental counseling) regarding automobile safety should include information about the potential dangers of heat stress, in fact, as these events are mostly unintentional, legislative efforts may be vain.

## Competing interests

The authors declare that they have no competing interests.

## Authors’ contributions

PF conceived of the study, RR and AC participated in its design and coordination and helped to draft the manuscript. FI and PL, contributed in data collection. FV, OC and VDV performed review of the literature. All authors read and approved the final manuscript.
